# Quantitative Proteomics Analysis Reveals XDH Related with Ovarian Oxidative Stress Involved in Broodiness of Geese

**DOI:** 10.3390/ani15020182

**Published:** 2025-01-11

**Authors:** Ning Zhou, Yaoyao Zhang, Youluan Jiang, Wang Gu, Shuai Zhao, Wanwipa Vongsangnak, Yang Zhang, Qi Xu, Yu Zhang

**Affiliations:** 1 Jiangsu Key Laboratory for Animal Genetic, Breeding and Molecular Design, Yangzhou University, Yangzhou 225009, China; znznzn20001231@163.com (N.Z.); yaoyaochn@outlook.com (Y.Z.); j25802580128@163.com (Y.J.); dx120210144@stu.yzu.edu.cn (W.G.); zhaoshuai73@163.com (S.Z.); zyang@yzu.edu.cn (Y.Z.); xuqi@yzu.edu.cn (Q.X.); 2 Key Laboratory for Evaluation and Utilization of Livestock and Poultry Resources (Poultry), Ministry of Agriculture and Rural Affairs, Beijing 100176, China; 3 Department of Zoology, Faculty of Science, Kasetsart University, Bangkok 10900, Thailand; wanwipa.v@ku.ac.th

**Keywords:** goose, XDH, oxidative stress, broody behavior

## Abstract

Studies have demonstrated significant alterations in ovarian oxidative stress levels, ovarian degeneration, and follicular atresia during the broody period in geese. The results of this study showed that during the broody period, geese exhibited degraded ovarian tissues, disrupted follicular development, a thinner granulosa cell layer, and lower levels of ovarian hormones E2, P4, and AMH. Antioxidant activity (GSH, CAT, SOD, T-AOC, and the content of H_2_O_2_) and the mRNA expression levels of antioxidant genes (GPX, SOD-1, SOD-2, CAT, COX-2, and Hsp70) were significantly higher in pre-broody geese compared to laying geese, while the expression of apoptosis-related genes (p53, Caspase-3, and Caspase-9) increased and the anti-apoptotic gene Bcl-2 decreased. Additionally, proteomic analysis identified 703 differentially expressed proteins (DEPs), primarily concentrated in the GO categories of the biological process (biological regulation, response to stimulus, etc.) and enriched in the KEGG pathways (PI3K-Akt signaling pathway, etc.). Among them, XDH was central to the regulatory network. Furthermore, Western blotting revealed higher expression of XDH in the ovaries of pre-broody geese than those of laying geese. Pearson correlation analysis indicated a significant correlation between XDH expression and oxidative stress markers in the ovaries of geese (r > 0.75). Overall, these results demonstrated that geese experience ovarian atrophy and remarkably increased oxidative stress during the broody period, suggesting that XDH may be a key driver of broodiness in geese.

## Simple Summary

The broody behavior of geese has seriously impacted egg production performance and hindered the development of the industry. However, the principal regulators and underlying physiological mechanisms of this biological process are not well understood. Consequently, this study aimed to identify key proteins by assessing the antioxidant levels in the ovary in conjunction with proteomic analysis to elucidate the mechanisms underlying geese broodiness and offer new insights to improve egg production performance.

## 1. Introduction

China is the world’s top producer and consumer of geese [1]. Geese are renowned for their exceptional adaptability, quick growth, high nutritional value, and low input needs, as they are herbivorous poultry. However, geese generally exhibit strong broodiness, which significantly reduces their laying period and overall laying performance, thereby hindering the development of the geese industry [2]. Additionally, broodiness in poultry is a trait with low heritability (h^2^ ≈ 0.116) [3]. Traditional reproductive regulation techniques can improve the laying performance of geese to some extent, but the effects are not significant [4]. Therefore, it is crucial to elucidate the molecular mechanisms underlying their reproductive biology. However, numerous studies have shown that the ovaries of geese experience strong oxidative stress during broodiness, which leads to follicular atresia and cessation of egg production [5,6,7,8,9]. Clarifying the key factors involved in ovarian oxidative stress-induced broodiness in geese will help to identify and select key molecules that inhibit oxidative stress to control or reduce broodiness in breeding geese.

Xanthine dehydrogenase (XDH) is one of the redox products of xanthine oxidoreductase (XOR); another is xanthine oxidase (XO). Both belong to the molybdenum-containing flavoprotein dehydrogenase family [10,11]. XDH is an important precursor of XO [12]. XDH/XO is an endogenous physiological regulator of cycloxygenase-2 (Cox-2) in the inflammatory system [13] and is also a key factor in the evolution and function of innate immunity [14]. Margolin and Behrman showed that XDH was active in the follicles and luteal tissues of the ovaries of rats [15]. Meneshian and Bulkley confirmed that reactive oxygen species (ROS) produced by XO are associated with clinicopathology such as ischemia, multi-system organ failure, and reperfusion injury [16]. Studies have also shown that the expression level of XDH increases significantly with the decidualization of endometrial stromal cells, which is an important process for establishing and maintaining pregnancy in early pregnancy, suggesting that XDH may be related to animal reproductive performance [17].

The appropriate concentration of ROS in the ovaries of female geese plays a crucial physiological role in follicular development and ovulation [18]. As part of the intra-follicular microenvironment, ROS are vital in the process of proliferation, differentiation, and apoptosis of granulosa cells [19]. Under oxidative stress, excessive ROS can lead to DNA mutation or cleavage, inactivation of important intracellular enzymes, and protein denaturation, which can trigger cell apoptosis, follicular atresia, and ovarian degeneration [20]. Studies have indicated that oxidative stress is a critical factor affecting animal reproductive performance and is a primary cause of ovarian degradation and follicular atresia in poultry [21]. Research in mammals, such as mice, has demonstrated that oxidative stress can decrease the number of follicles at all stages of ovarian development and impair ovarian function; this process can be alleviated by the antioxidant melatonin [22]. Oxidative stress can also induce apoptosis of oocytes, a phenomenon that can be inhibited by catalase [23]. Both in vivo and in vitro oxidative stress have detrimental effects on oocyte quality, apoptosis, and autophagy of granulosa cells and disrupt the ovulation process, ultimately resulting in follicle degradation, atresia, and decreased egg production in poultry [24,25].

Current studies suggest that the regulation of oxidative stress to follicular development or atresia is mainly through mediating signaling pathways, such as FasL-Fas, Cyt-c-Bax/Bcl2-Caspase, and Nrf2-Keap1, among others. Additionally, antioxidant-related genes such as SOD1 and GSH have been identified to play important roles in preventing follicular apoptosis [26]. However, most research has primarily focused on the mechanism of oxidative stress mediating follicular atresia caused by environmental stressors such as heat stress, mycotoxins, and heavy metals [27]. Recently, Ye et al. conducted a hypothalamic transcriptome analysis on broodiness in Muscovy ducks, revealing cyclical mechanisms of reproductive function conversion affected by oxidative stress [28]. Furthermore, studies have found that the level of oxidative stress in the ovary changes significantly from the laying period to the pre-broody period, accompanied by ovarian degeneration and follicular atresia [29,30]. The antioxidant vitamin E can inhibit oxidative stress and alleviate the autophagy and apoptosis of granulosa cells. However, the key proteins and pathways involved in this biological process are still unclear.

Therefore, this study focused on Zhedong White geese, which exhibit strong broodiness. We compared the changes in ovarian oxidative stress levels between the laying and pre-broody periods, explored the proteomic changes of ovarian tissues during broodiness, and identified key proteins and pathways involved in ovarian oxidative stress-induced broodiness in geese. Our findings aim to provide critical targets for controlling or reducing broodiness by inhibiting oxidative stress. The results will not only enhance the theoretical understanding of the physiological mechanism of broodiness in geese but also offer new insights for improving their egg production performance.

## 2. Materials and Methods

### 2.1. Ethics Statement

The Yangzhou University (Jiangsu, China) Institutional Animal Care and Use Committee (IACUC) gave its approval to all animal-related procedures during this research (Approval Date: December 2020). These procedures also adhered to the Jiangsu Administration Rules for Laboratory Animal Use. The environment and living conditions of the geese met the standards of China’s Laboratory Animal Requirements of Environment and Housing Facilities https://www.standardsofchina.com/standard/GB14925-2010 (accessed on 31 December 2023).

### 2.2. Experimental Animals and Sample Collection

Twelve ten-month-old, female Zhedong White geese were selected from the National Waterfowl Conservation Center (Jiangsu, China). This flock of geese had free access to a controlled diet (Table 1). Geese were monitored using a video camera (Jindun, 720p, Nanjing, China) and were identified to be three each in the laying, pre-broody, broody, and post-broody periods, as detailed previously [31]. Briefly, when geese are laying eggs, we classify them as being in the laying period. Additionally, when geese exhibit nest-seeking behavior and repeatedly use the husks to construct nests, we classify them as being in the pre-broody period. The geese begin the broody stage if they exhibit continuous half-closed-eye slumber, deny mounting from the gander, and consume significantly more food and liquids than they did before the broody period, which can last up to 22 h per day. Following that, the geese gradually increase their drinking and feeding habits in comparison to the broody period, and they occasionally leave the nest. We classify this period as the post-broody period. After identifying the geese in the appropriate phases, isoflurane was used to anesthetize and euthanize them. The ovaries were rapidly collected and divided into four parts for further analysis: ELISA assay, quantitative real-time PCR (qPCR), Western blotting, and proteomics sequencing, respectively.

### 2.3. Morphological Observation of Ovarian Tissues

Ovarian morphology was observed, and the number of small yellow follicles in each ovary was counted. Following a 24-h fixation in 4% paraformaldehyde (PFA), the tissues of the ovarian follicle were dried, cleaned, paraffin-embedded, and sectioned at 6–8 μm for placement on glass slides. Then, we stained the slides with hematoxylin and eosin (H&E) and then captured digital photographs for examination under a microscope (Nikon Corp, Tokyo, Japan). Additionally, a 1 mm^3^ sample of ovarian follicle tissue was also promptly removed and put in an electron microscope fixative to create ultrathin sections for transmission electron microscopy. The representative region of the ultrathin section was observed with a Hitachi 7800 electron microscope (Japan) for image analysis.

### 2.4. Detection of Antioxidant Activity and Reproductive Hormone Levels of Ovaries by ELISA Assay

First, 100 mg of tissue was weighed into a homogenization tube and 900 µL of physiological saline was added. A 10% tissue homogenate was created by homogenizing the tissue at ice-cold temperatures. The supernatant was then extracted by centrifuging the tissue for ten minutes at 2500 rpm. Physiological saline was added to the supernatant until the desired sample concentration was achieved. Subsequently, assay kits for glutathione (GSH), catalase (CAT), superoxide dismutase (SOD), total antioxidant capacity (T-AOC), and hydrogen peroxide (H_2_O_2_), provided by Nanjing Jiancheng (Nanjing, China), were used to detect oxidative contents of the ovaries. The separated tissue samples were tested for reproductive hormone contents. Using enzyme-linked immunosorbent assay (ELISA) kits (Nanjing Jiancheng), the concentrations of progesterone (P4), estradiol (E2), and anti-Müllerianhormone (AMH) were determined with an Infinite M200 Microplate reader (Tecan, Männedorf, Switzerland), following the manufacturer’s instructions.

### 2.5. Immunohistochemistry

Ovary tissues of geese were immediately collected, fixed with 4% paraformaldehyde, and embedded in paraffin for immunohistochemical staining. Tissue sections (5 µm thick) were incubated with primary antibodies against Bcl-2, Caspase-3, and Hsp70 overnight at 4 °C. Following that, secondary antibodies (anti-rabbit IgG, Abcam, Cambridge, UK) were incubated for one hour at 37 °C. The average integrated optical density (IOD) of cells positively expressing Bcl-2, Caspase-3, and Hsp70 was measured in six randomly selected regions per sample using Image-Pro Plus 5.1 image analysis software.

### 2.6. Quantitative Real-Time PCR

The TRIzol (Invitrogen, Carlsbad, CA, USA) kit was used to isolate total RNA, followed by chloroform extraction. Following the manufacturer’s instructions, a FastQuant RT kit (with gDNase) (Tiangen, Beijing, China) was used to synthesize the cDNA strand from 1 g of total RNA. The expression levels of different genes were analyzed using a gene-specific primer set with a Biosystems 7500 Real-Time PCR device (Thermo Fisher Scientific, Waltham, MA, USA), using β-actin as a reference control. The comparative CT method (2^−ΔΔCt^ method) was employed for analysis. All primers used in this study were synthesized by Tsingke Biotechnology Co., Ltd. (Beijing, China) and are listed in Table 2.

### 2.7. Western Blotting

Proteins were deposited onto the PVDF membrane following gel electrophoresis using 10% SDS-PAGE. The PVDF membranes were then incubated in 5% skim milk (diluted in Tris Buffered Saline with Tween-20 (TBST)) for 2 h and subsequently incubated at 4 °C overnight with diluted primary antibodies (XDH Rabbit mAb, ABclonal#A22335; β-actin Rabbit mAb, ABclonal#AC026, Wuhan, China). After washing with TBST three times, the PVDF membrane was incubated with goat anti-rabbit IgG (BIOMIKY, Shanghai, China) for 90 min. The chemiluminescence imaging system 398 (UVitec, Cambridge, UK) was used to visualize the protein blots, and ImageJ1.54d software (National Institutes of Health, Bethesda, MD, USA) was used to quantify the results.

### 2.8. Protein Extraction, Trypsin Digestion, and TMT Proteomic Labeling

Each group comprised three biological replicates (n = 3), three in the laying period, and three in the pre-broody period. A total of 6 UF (3 UF/group) were suspended in lysis buffer, and the supernatants were collected by centrifuging the respective suspensions for 20 min at 12,000× *g*. The Pierce™ BCA Protein Assay Kit (Thermo Fisher Scientific, USA) was used to measure the amount of protein in the supernatant. Trypsin was used to digest the sample protein and extract the digested protein peptides in accordance with the FASP protocol [32]. After trypsin digestion, samples were desalted by C18 cartridges (Sigma, St. Louis, MO, USA) and then vacuum-dried. As directed by the kit (Thermo Fisher Scientific, USA), the peptides (100 μg) were reconstituted and processed using the TMT16plex™ Isobaric Label Reagent Set.

### 2.9. HPLC Fractionation and LC-MS/MS Analyses

Reverse-phase HPLC with a Waters XBridge Peptide BEH C18 column was used to separate the peptide mixtures with Nanospray FlexTM ion source (2.0 kV). The mass spectrometer was used to gather the mass spectrometry (MS) data. With a resolution of 120,000 (at 200 *m*/*z*), the MS1’s complete scanning range was 350–1800 *m*/*z*. parent ions with the TOP 40 ionic strengths from the MS1 full scan were chosen for fragmentation by high-energy collision dissociation at a resolution of 30,000 (200 *m*/*z*) for the second-stage mass spectrometry (MS2) scans [33].

### 2.10. Database Search and Bioinformatics Analysis

For the database search, the raw data were deposited in the Integrated Proteome Resources (iProX) of the ProteomeXchange under the bio-project number PXD055319 and the reverse library was added to calculate the false discovery rate (FDR) caused by random matches. Proteome Discoverer (version 2.4.1.15) was used to analyze mass spectrometry RAW data for quantitative assessment. In this study, the identified proteins were annotated and analyzed for enrichment using eggnog-mapper software (v2.0) and the eggNOG database for gene ontology (GO) annotation and enrichment analysis, the PfamScan tool and the InterPro database for protein domain annotation and enrichment analysis, and the Kyoto Encyclopedia of Genes and Genomes (KEGG) pathway database for protein pathway annotation and enrichment analysis [29]. A two-tailed Fisher’s exact test was used to compare the enrichment of the differentially expressed proteins for each category to all identified proteins; a *p*-value of less than 0.05 was considered significant. The Jingjie Bioinformatics Cloud Platform (http://www.ptmbiolab.com accessed on 31 December 2023) was then used to perform protein-protein interaction and motif studies in order to discover important regulation-changed proteins under particular experimental settings.

### 2.11. Parallel Reaction Monitoring (PRM) Validation of Goose Ovaries Proteomics Results

The samples utilized for omics PRM verification were identical to those described in Section 2.8. PRM verification of 10 target proteins was conducted by Jingjie Biotechnology Co., Ltd (Hangzhou, China). The methodology is outlined as follows: Peptide information suitable for PRM analysis was imported into Xcalibur 4.3 software to establish the PRM method. Approximately 1 μg of peptide from each sample was combined with 20 fmol of standard peptide (PRTC: ELGQSGVDTYLQTK) for detection. Chromatographic separation was achieved using an HPLC system. The samples separated by HPLC were analyzed via PRM mass spectrometry with a Q-Exactive HF mass spectrometer (Thermo Scientific, Waltham, MA, USA). Data analysis for PRM was performed using Skyline software (version 3.5.0).

### 2.12. Statistical Analysis and Data Available

Data comparisons between behavioral groups (laying, pre-broody, broody, and post-broody period) were analyzed using one-way ANOVA, which is a completely randomized design. The LSD method was applied to test differences between groups using IBM SPSS Statistics 22.0 software (Armonk, NY, USA). All data were presented as mean ± standard deviation (SD), and results with a *p*-value ≤ 0.05 were considered statistically significant. Fisher’s exact test was used for two-tailed testing of protein enrichment levels for all identified proteins to determine differences in protein expression. Pearson correlation analysis was carried out to characterize the associations between the expression levels of DEPs and the activity of antioxidant stress-related enzymes.

## 3. Results

### 3.1. Morphological and Histological Characteristics of Ovaries in Laying, Pre-Broody, Broody, and Post-Broody Periods

**Figure 1:**
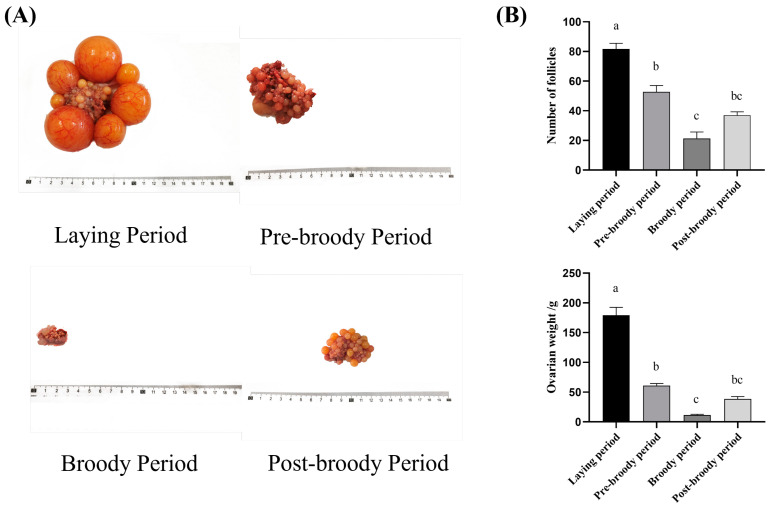
Morphological observation of ovaries in laying, pre-broody, broody, and post-broody geese. General appearances (**A**) and statistical analysis of number and weight (**B**) of ovaries in laying, pre-broody, broody, and post-broody geese. The error bars represent the standard error (SE) of three replicates. Different lowercase letters indicate significant differences (*p* < 0.001).

To understand the morphological changes in the ovaries of Zhedong White geese during the laying, pre-broody, broody, and post-broody periods, anatomical observations were conducted. The findings showed that the ovaries of broody geese had much smaller volumes, weights, lengths, and widths than those of laying geese (Figure 1A,B). Compared to laying geese, the follicles in broody geese exhibited severe degeneration, atresia, and disruption of the developmental hierarchy, with pre-ovulatory follicles absent. The number of follicles in broody geese was fewer than in laying geese (Figure 1B). Follicles in the ovaries of broody geese showed varying degrees of collapse and deformation, along with decreased elasticity; both the cortex and medulla of the ovaries were shrunken in broody geese.

**Figure 2:**
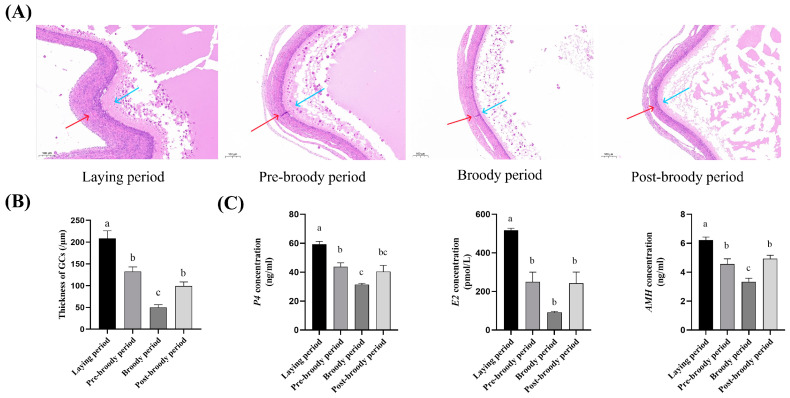
HE staining results and comparison of the levels of reproductive hormones. (**A**) HE staining results of small yellow follicles of laying, pre-broody, broody, and post-broody geese. Note: The blue arrows represent the granulosa cell layer and the red arrows represent the membrane layer. The ruler is 100 μm. (**B**) The thickness of follicle granulosa cell layers in laying, pre-broody, broody, and post-broody periods. (**C**) Comparison of the levels of reproductive hormones of ovaries during different periods. The error bars represent the SE of three replicates. Different lowercase letters indicate significant differences (*p* < 0.001).

HE staining showed that in atretic follicles of the broody period, the nuclei of the granulosa cells were pyknotic, the connections between the granulosa cells and the parietal cells were loose, some areas were exfoliated inward, the cytoplasm was concentrated to varying degrees, and vacuoles appeared. The oocyte shrank, the granulosa cell layer separated from the membrane layer, and there was a rupture of the theca, granulosa cells layer, and vitelline membrane. The findings revealed an uneven distribution of GCs and a thinner GC layer in the follicles of broody geese (Figure 2A,B). To further determine the changes in reproductive hormones during broodiness, the concentrations of major hormones were measured. Compared with the laying period, the concentrations of P4, E2, and AMH of ovarian tissue during the broody period were significantly decreased (Figure 2C).

### 3.2. Antioxidant Activity of Ovaries Among Laying, Pre-Broody, Broody, and Post-Broody Periods

**Figure 3:**
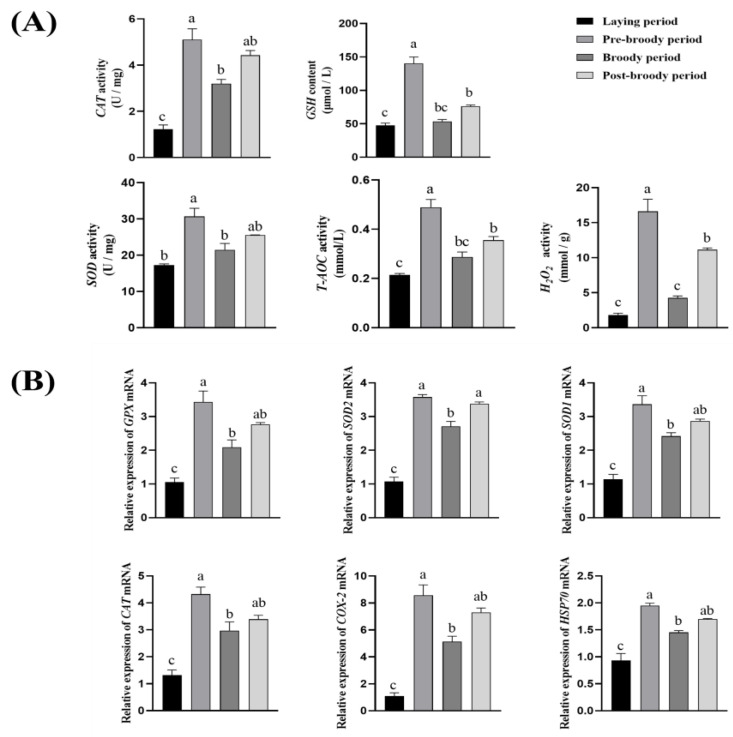
Antioxidant activity of ovaries in laying, pre-broody, broody, and post-broody geese. (**A**) Differences in levels of enzymes related to oxidative stress (CAT, SOD, T-AOC, GSH, and the content of H_2_O_2_) in ovaries during different periods (n = 3). (**B**) Differences in levels of genes related to oxidative stress (GPX, SOD-1, SOD-2, CAT, COX-2, and Hsp70) in ovaries during different periods (n = 3). The error bars represent the SE of three replicates. Different lowercase letters indicate significant differences (*p* < 0.001).

To explore the changes of oxidative stress levels in the ovarian tissue of Zhedong White geese during broodiness, the activities of CAT, SOD, T-AOC, GSH, and the content of H_2_O_2_ in the ovarian tissue were measured. The results showed that, compared with the laying period, the activities of CAT, SOD, T-AOC, GSH, and the content of H_2_O_2_ were significantly increased during the pre-broody period (Figure 3A). To further determine the changes in oxidative stress levels in ovarian tissue during broodiness, RT-qPCR was used to detect the relative mRNA expression levels, superoxide dismutase 2 (SOD-2), glutathione peroxidase (GPX), catalase (CAT), cyclooxygenase-2 (COX-2), superoxide dismutase 1 (SOD-1), and heat shock protein 70 (Hsp70). The results indicated that, in terms of mRNA expression levels, the expression levels of GPX, SOD-1, SOD-2, CAT, COX-2, and Hsp70 during the pre-broody period were considerably greater than those in the laying period (Figure 3B).

### 3.3. Apoptosis Level of Ovaries During Laying, Pre-Broody Broody, Broody, and Post-Broody Periods

**Figure 4:**
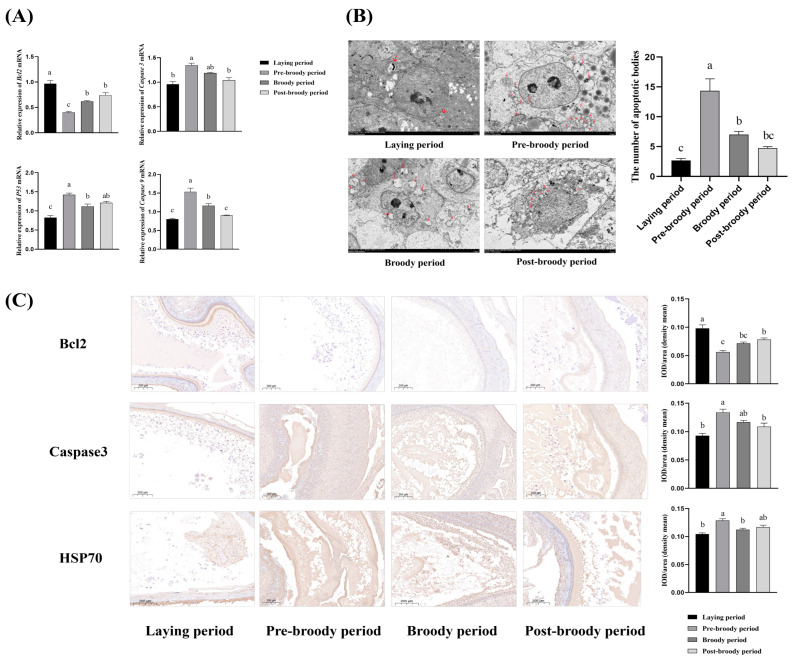
Apoptotic detection of follicles in ovaries during laying, pre-broody, broody, and post-broody periods. (**A**) Differences in levels of apoptosis in ovaries among laying, pre-broody, broody, and post-broody periods (n = 3). (**B**) Electron microscopy to observe the difference in the number of apoptotic bodies in granulosa cells of follicles among laying, pre-broody, broody, and post-broody periods. Arrows represent apoptotic bodies. (**C**) Immunohistochemical staining of Bcl-2, Caspase-3, and Hsp70 and their quantitative analysis based on the mean density. The error bars represent the SE of three replicates. Different lower-case letters indicate significant differences (*p* < 0.001).

To explore the difference in the level of apoptosis of the granulosa cell layer among laying, pre-broody, broody, and post-broody geese, we used RT-qPCR to detect the expression differences of genes related to apoptosis across the four periods. The relative mRNA expression levels of Caspase-3, Caspase-9, and p53 in pre-broody geese were significantly increased compared with laying geese, whereas the opposite trend was observed for Bcl-2 expression (Figure 4A). Observation of the follicular granulosa layer cells during the laying, pre-broody, broody, and post-broody periods showed that, compared with the laying period, the number of apoptotic bodies was significantly higher in the pre-broody period (Figure 4B). Immunohistochemistry detected the expression of Bcl-2, Caspase-3, and Hsp70 in follicles among the laying, pre-broody, broody, and post-broody periods. The results indicated that the expression of Caspase-3 and Hsp70 in the pre-broody period was significantly higher than that in the laying period, while the expression for Bcl-2 showed the opposite trend (Figure 4C).

### 3.4. Quantitative Proteomics Analysis of Ovaries Between Laying and Pre-Broody Periods

**Figure 5:**
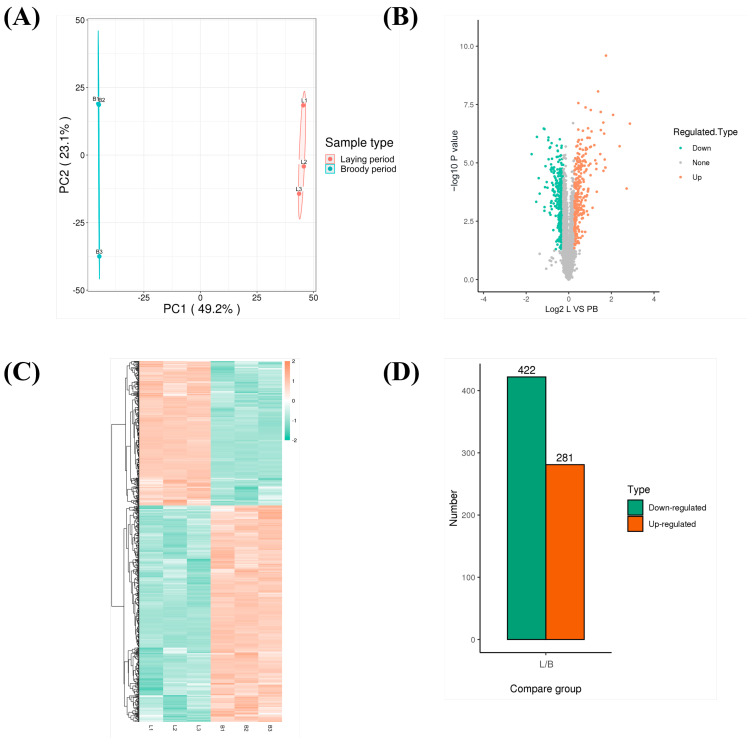
Screening of DEPs in ovaries of laying and pre-broody geese. (**A**) PCA of proteomic data. (**B**) Volcano plot of laying period/pre-broody period group. The x-axis indicates log_2_ (fold change), while the y-axis corresponds to the mean expression value of log_10_ (*p*-value). (**C**) Heat map of DEPs. (**D**) The number of up-regulated and down-regulated proteins between ovaries of laying and pre-broody geese. (L, laying period; PB, pre-broody period).

**Figure 6:**
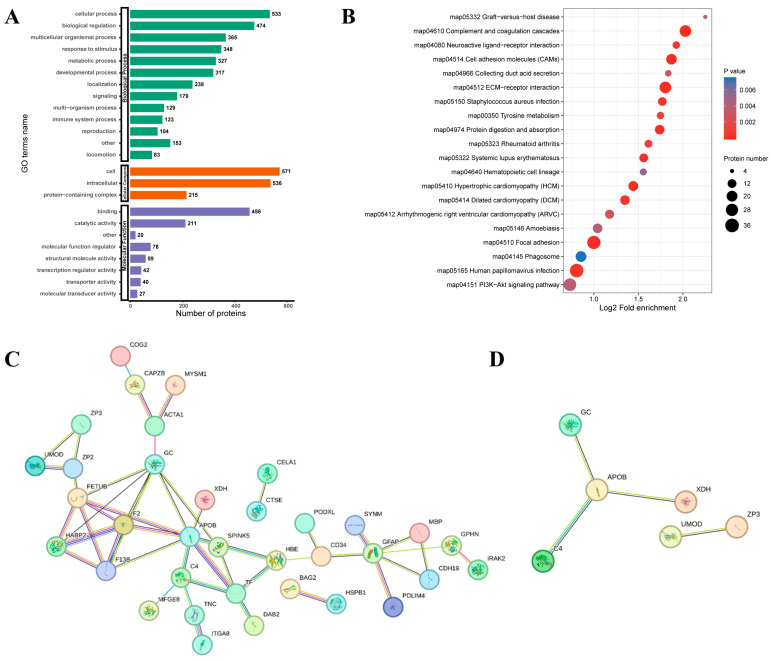
Functional enrichment and interaction analysis of DEPs in ovaries of laying and pre-broody geese. (**A**) GO enrichment analysis of DEPs. (**B**) Bubble charts showing KEGG pathway enrichment of DEPs. (**C**) Protein-protein interaction network of DEPs. (**D**) The key node proteins of the protein-protein interaction network.

To explore the changes in the ovarian proteome in Zhedong White geese between the laying period and the pre-broody period, TMT (tandem mass tags) labeling technology combined with liquid chromatography-tandem mass spectrometry (LC-MS/MS) was used to screen DEPs. A total of 703 DEPs were identified. Compared with the laying period, 422 up-regulated proteins including XDH, an inhibitor of kappa B kinase (IKKA), a cluster of differentiation 44 (CD44), and Laminin (LN) were identified, while 281 down-regulated proteins including PH domain leucine-rich repeat protein phosphatase (PHLPP), extracellular matrix (ECM), integrin alpha (ITGA), integrin beta (ITGB), and major histocompatibility complex II (MHC-II) were identified in the pre-broody geese (Figure 5). GO classification revealed that the DEPs were mainly concentrated in the categories of cellular process, biological regulation, multicellular organismal process, and response to stimulus in biological process (Figure 6A). Meanwhile, functional enrichment analysis of DEPs showed that they were mainly enriched in the KEGG signaling pathways, which were mainly enriched in the cell adhesion molecules (CAMs), PI3K-Akt signaling pathway, ECM-receptor interaction, phagosome, protein digestion and absorption, tyrosine metabolism, and neuroactive ligand-receptor interaction. (Figure 6B). The PPI network identified the key regulatory proteins in the DEPs (Figure 6C). XDH, apolipoprotein b (APOB), GC vitamin D binding protein (GC), complement 4 (C4), zona pellucida glycoprotein 3 (ZP3), and uromodulin (UMOD) were identified as key node proteins (Figure 6D).

### 3.5. Parallel Reaction Monitoring (PRM) Analysis of DEPs Between Laying and Broody Periods

**Figure 7:**
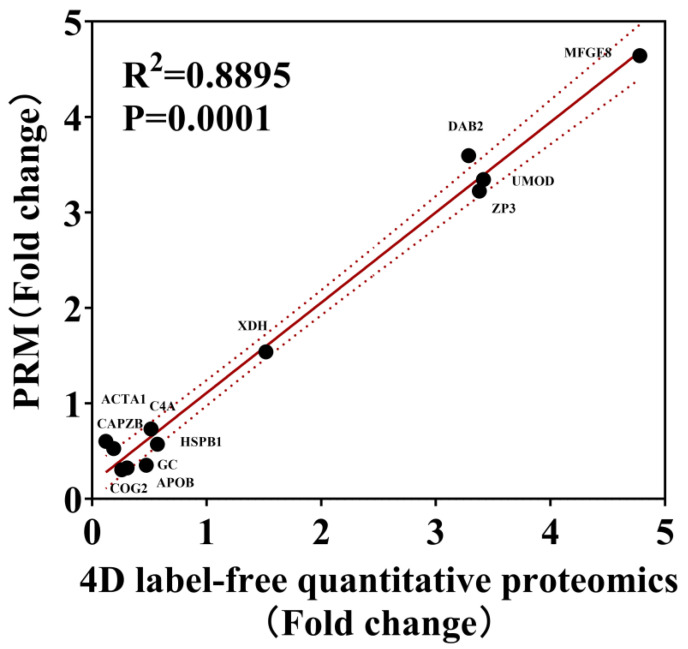
Regression analysis of the expression of DEPs detected by PRM and label-free proteomics. The expression trends of 12 target proteins were compared with the label-free sequencing results.

Based on the proteomics results of geese ovaries, 12 proteins were randomly selected from all DEPs to verify their expression levels using PRM analysis. The selected target proteins (COG2, APOB, GC, CAPZB, HSPB1, ACTA1, C4A, MFGE8, XDH, DAB2, ZP3, and UMOD) were consistent with the expression trend observed in the label-free sequencing results, and the R^2^ was 0.8895, confirming the reliability of the proteomics findings (Figure 7).

### 3.6. XDH Promotes Ovarian Oxidative Stress in Mediating Broodiness of Geese

**Figure 8:**
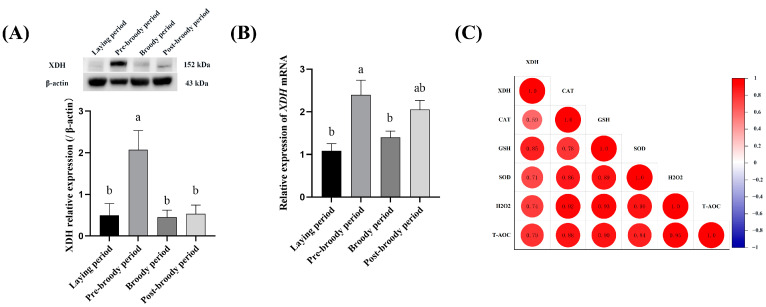
XDH contributes to the broodiness of geese by promoting ovarian oxidative stress. (**A**) Relative expression levels of XDH protein in the ovaries of laying, pre-broody, broody, and post-broody geese. (**B**) Differences in levels of XDH mRNA in ovaries among different periods (n = 3). (**C**) Correlation between differential expression of XDH and oxidative stress-related indicators in ovaries of broody geese. The error bars represent the SE of three replicates. Different lowercase letters indicate significant differences (*p* < 0.05).

Compared with the ovarian tissues of laying geese, the expression of XDH was significantly increased in the ovarian tissue of pre-broody geese, suggesting that XDH may be involved in the occurrence of broodiness in geese. Furthermore, the results revealed higher expression of XDH in follicular granulosa cells of pre-broody geese compared to those of laying geese (Figure 8A). To further determine the difference in expression levels of XDH during different periods, RT-qPCR was used to detect the relative mRNA expression levels. The results indicated that the mRNA expression levels of *XDH* during the pre-broody period were considerably increased than those in the laying period (Figure 8B). The Pearson correlation coefficient was employed to analyze the correlation between XDH expression and oxidative stress-related indicators, namely CAT, GSH, SOD, H_2_O_2_, and T-AOC, in the ovaries of broody geese. The results revealed a highly significant positive correlation overall (r > 0.75, Figure 8C). This indicates that the up-regulation of XDH expression in the ovaries of broody geese is strongly correlated with the oxidative stress level in the ovarian tissue.

## 4. Discussion

When birds enter the broody phase, it leads to significant changes in the oxidative stress level of the body, resulting in ovarian degeneration, follicular atresia, and programmed death of follicular granulosa cells [7]. Identifying and screening the key factors involved in this biological process will help uncover the mechanism of broodiness in geese and aid in developing molecular markers to control it, potentially boosting egg production. In this study, we explored the morphological and molecular shifts in Zhedong White geese ovaries across laying, pre-broody, broody, and post-broody periods. Notably, oxidative stress was elevated in pre-broody geese, as evidenced by increased antioxidant enzyme activities and higher H_2_O_2_ levels [30]. The broody geese exhibited reduced ovarian dimensions and follicular degeneration, with a significant decrease in follicle count and severe atresia. Apoptosis was also upregulated, with increased expression of pro-apoptotic genes and a rise in apoptotic bodies. However, given the restriction of q-PCR sample size, the statistical robustness and generalizability of oxidative stress levels and apoptosis levels may be compromised, thereby limiting the extrapolation of subsequent experimental results and research conclusions to some extent. Proteomics identified 703 DEPs, with up-regulation of proteins such as XDH correlating strongly with increased oxidative stress, suggesting a key role in broodiness. These findings underscore the intricate changes in ovarian physiology associated with broodiness, highlighting the importance of oxidative stress in reproductive transitions.

Oxidative stress is closely linked to broodiness in birds, potentially playing a critical role in the physiological shifts that occur during the brooding phase [7,34]. A certain amount of ROS in the ovary plays an important physiological role in its development and ovulation. However, when ROS levels are excessively elevated, intense oxidative stress damages biomolecules in all types of cells, leading to impaired follicular development and accelerated oocyte senescence. Elevated oxidative stress levels have been observed in the ovaries of broody hens, which can lead to degeneration, follicular atresia, and apoptosis of granulosa cells [35,36,37]. In this study, we monitored geese and identified four distinct behavioral periods using camera surveillance: laying, pre-broody, broody, and post-broody. These four periods comprise the entire reproductive cycle. The levels of oxidative stress were significantly increased in the pre-broody period, leading to a reduced antioxidant capacity of ovarian tissues, apoptosis of granulosa cells, follicular atresia, and ovarian atrophy, which ultimately resulted in the cessation of egg production. In the study of mechanisms of broodiness in Muscovy ducks, oxidative stress-related genes such as SOD3, NOS2, TH, TPH2, and SESN1 were discovered [28]. One possible explanation is that oxidative stress may contribute to the reproductive costs during broodiness [38,39]. In this study, our results demonstrated that, compared with the laying period, the activity of antioxidant indices (CAT, SOD, T-AOC, GSH, and H_2_O_2_ content) was significantly increased in the pre-broody period. The relative mRNA expression levels of antioxidant genes (GPX, SOD-1, SOD-2, CAT, COX-2, and Hsp70) were significantly elevated in the pre-broody period. Our study provided stronger evidence for the hypothesis that broodiness was closely related to the oxidative stress level of the ovary in geese.

In this study, functional enrichment analysis of DEPs showed that they were mainly concentrated in the GO categories of biological process (cellular process, biological regulation, response to stimulus) and enriched in the KEGG pathways, which were PI3K-Akt signaling, CAMs, ECM-receptor interaction, phagosome, protein digestion and absorption, tyrosine metabolism, and neuroactive ligand-receptor interaction. Cell survival, differentiation, and proliferation all depend on the PI3K-Akt signaling pathway [40], and Akt/PKB can deactivate pro-apoptotic proteins, including Caspase-9 and Bad [41,42]. The PI3K/Akt signaling pathway is one of the important pathways in the regulation of cell proliferation, mainly by promoting cell proliferation to resist apoptosis. Magnolia may improve antioxidant enzyme activity and expression of related genes via the PI3K-Akt pathway to alleviate oxidative stress [43]. Several proteins involved in the PPI network of DEPs are related to oxidate stress. HSPB1 can exert strong antioxidant activities by maintaining the levels of reduced GSH and stabilizing the mitochondrial membrane [44,45]. Milk fat globule epidermal growth factor 8 (MFG-E8) is involved in several biological functions, such as lowering oxidative stress and apoptosis [46]. XDH is located in the center of the PPI network and is significantly correlated with levels of oxidative stress indicators (GSH, CAT, SOD, T-AOC, and H_2_O_2_). XDH, a key enzyme in purine metabolism, contributes to both the generation of ROS and the mitigation of oxidative stress [47]. While its catalytic activity can lead to the production of ROS and subsequent cellular damage, it can also be a target for antioxidant therapies to reduce oxidative stress [48,49]. This dual role of XDH underscores its significance in balancing cellular redox states and its potential as a therapeutic target in conditions involving oxidative stress. It can be concluded that XDH plays a significant role in maintaining the cellular redox state. Furthermore, it may have the potential to serve as a target for regulating nesting behavior under physiological conditions of oxidative stress in geese. In ovaries cultivated with DEHP, the expression of the gene XDH, which is linked to oxidative stress, was markedly increased [50]. C1QBP enhances hypoxanthine catabolism and elevates the apoptosis of RCC cells by regulating XDH-mediated ROS production [51]. By enhancing gene expression linked to antioxidant enzymes and SOD and GPX activities to improve antioxidant capacity, exogenous spermidine reduces the death of granulosa cells [52]. This suggests that intervening with XDH to relieve oxidative stress levels could potentially prolong the laying cycle. Next, we will supplement the XDH inhibitor in the pre-broody period based on broody behavior characteristics and investigate its effect on broodiness and egg production performance in geese.

## 5. Conclusions

The ovaries of geese undergo significant alterations during the four periods from the laying period to the post-broody period, gradually shrinking and degenerating, especially during the broody period. Compared with the laying period, the concentrations of P4, E2, and AMH in ovarian tissue during the broody period were significantly decreased, which was consistent with the changing trends in ovarian traits. The levels of oxidative stress and apoptosis in the pre-broody period were significantly higher than those in the laying and broody periods. Proteomics analysis revealed a total of 703 DEPs obtained during broodiness in geese. These DEPs were predominantly associated with biological processes such as cellular activities and responses to stimuli and were enriched in pathways such as PI3K-Akt signaling and CAMs. The PPI network analysis revealed that XDH was located at the center of the interaction network. Western blotting detected a higher expression of XDH in the ovaries of pre-broody geese compared to laying geese, which was found to be significantly correlated with levels of oxidative stress indicators (GSH, CAT, SOD, T-AOC, and H_2_O_2_), as determined by Pearson (r > 0.75). The results indicate the involvement of XDH in ovarian oxidative stress associated with geese broodiness, providing candidate targets for breeding selection aimed at regulating brooding behavior and enhancing laying performance from the perspective of physiological oxidative stress levels.

## Figures and Tables

**Table 1:** Composition and nutrient content of the experimental diet.

Item	Content (%)	Item	Content (%)
Corn	35	AME (MJ/kg)	10.82
Soybean meal	15	CP (%)	15.15
Alfalfa meal	5	Crude fiber (%)	5.24
Rice hull	10	Calcium (%)	1.17
Barley	10	Total phosphorus (%)	0.64
Wheat	15		
Bran	5		
Vitamin and trace mineral mix	5		

**Table 2:** Primers used for real-time qPCR of genes.

Gene	Nucleotide Sequence (5′~3′)	Length/bp	Tm/°C
*Bcl-2*	F: ATGACCGAGTACCTGAACCG R: GCTCCCACCAGAACCAAAC	155	60
*Caspase-3*	F: CTGGTATTGAGGCAGACAGTGG R: CAGCACCCTACACAGAGACTGAA	158	60
*Caspase-9*	F: TTCCAGGCTCTGTCGGGTAA R: GTCCAGCGTTTCCACATACCA	150	60
*p53*	F: ACCGGTGAGGGGACGAGAA R: GAGCGGCGGCGAGTTGGAG	190	60
*β-actin*	F: GAGAAATTGTCCGTGACATCA R: CCTGAACCTCTCATTGCCA	152	60
*GPX*	F: GCAAGGGGTACAAGCCCAACT R: GATGATGTACTGCGGGTTGGTC	149	60
*SOD-1*	F: CACCTGCTGTAACCATTCTTAGT R: GGCTCCTCATCTTCCAAACC	137	60
*SOD-2*	F: CCTGACCTGCCGTATGACTATG R: ACCTGAGCTGTAACATCACCTTTT	161	60
*CAT*	F: ATACAGTTCGTGACCCTCG R: CCAGAAGTCCCATACCAT	188	60
*COX-2*	F: CGATGAACCAGACCTCACCC R: TCTGGGGTGGGCACTATGTA	111	60
*Hsp70*	F: GGGAGACAAGTCCGAGAATGTGC R: GTTTGGTGGGAATGGTGGTGTTAC	128	60
*XDH*	F: GTAGGGAACACGGAAGTGGG	126	60
	R: GCCAAAGGTGACCCCTGTTT		

## Data Availability

All data generated or analyzed during this study are included in this published paper.
